# Furloughs, Teleworking and Other Work Situations during the COVID-19 Lockdown: Impact on Mental Well-Being

**DOI:** 10.3390/ijerph18062898

**Published:** 2021-03-12

**Authors:** Israel Escudero-Castillo, Fco. Javier Mato-Díaz, Ana Rodriguez-Alvarez

**Affiliations:** 1 Department of Applied Economics, University of Oviedo, 33007 Oviedo, Spain; jmato@uniovi.es; 2 Department of Economics, University of Oviedo, 33007 Oviedo, Spain; ana@uniovi.es

**Keywords:** precarious employment, health outcomes, work-family issues

## Abstract

As a consequence of the Spring 2020 lockdown that occurred in Spain due to the COVID-19 pandemic, many people lost their jobs or had to be furloughed. The objective of this study is to analyse the influence of the latter changes in labour market status on psychological well-being. For this purpose, an ad-hoc questionnaire featuring socio-demographic and mental health criteria was created. Granted that the pandemic can be viewed as an exogenous shock, the bias caused by the bidirectional problems between the work situation and mental well-being can be tackled. Results indicate that the lockdown exerted a greater negative effect on the self-perceived well-being of unemployed and furloughed persons than on those in employment. Moreover, among those in continuous employment, teleworkers experienced a lesser degree of self-perceived well-being post lockdown as compared to those people remaining in the same work location throughout the COVID-19 crisis. Finally, the lockdown provoked worse effects on the self-perceived well-being of women as compared to men, a result that appears to be related to gender differences in household production. In conclusion, these results could be especially relevant given that the evolution of the pandemic is having ongoing effects on employment and, therefore, on the mental health of workers.

## 1. Introduction

Between mid-March and early May 2020, Spain experienced a general lockdown due to the consequences of the COVID-19 pandemic. Coinciding with other world economies, the unprecedented situation strongly impacted the economy as well as employment rates [1]. Thus, many people lost their jobs due to the pandemic or had to be furloughed while efforts were made to bring the pandemic under control.

The objective of this article is to analyse the relationships occurring between each work situation and individual PWB resulting from the COVID-19 pandemic in Spain. For this purpose, an ad-hoc questionnaire featuring socio-demographic and mental health items was created. Given the conditions of confinement, the information was obtained through an online directory that could be accessed via a link.

The COVID-19 economic lockdown resulted in changes within the labour market affecting most of the working-age population [2]. Apart from those who directly lost their jobs or were furloughed, other workers switched to teleworking, and yet another group maintained their usual presence-based working conditions subject to the restrictions imposed by the health authorities. In total, almost 2.7 billion workers around the world were affected by the exceptional measures implemented in order to control the health crisis [3]. In Spain, the sudden arrival of the pandemic generated transitions toward a variety of work situations: teleworking, temporary unemployment, ordinary unemployment or regular altered employment (e.g., health services, supermarkets, distribution and other essential services to name but a few).

Health economics literature has for some time studied in depth the relationship existing between different work situations and the propensity for impaired psychological well-being (PWB).

The added value of this article is twofold. Firstly, the lockdown of the country and the subsequent confinement permitted exceptional conditions for analysing the influence of labour market status on mental health. Thus, the survey was designed to first distinguish between how people were employed prior to the onset of COVID-19 pandemic and how they became unemployed after confinement. This exceptional situation offers a unique opportunity to analyse the influence of the labour situation on psychological well-being, since the external shock reduces the double causality between both variables [4]. Secondly, the significant growth of temporary labour market situations, such as furloughs and teleworking, allowed an extension of the analysis to include the extent by which well-being may change as a result of specific employment and unemployment conditions.

## 2. Literature Review

The current COVID-19 pandemic has initiated a global health crisis. In addition to the serious consequences that this virus derived for physical health in an important part of the population, its advancement could also be affecting the psychological well-being of individuals.

The increase in anxiety and stress may be attributable to a variety of factors. Firstly, people have been exposed to a permanent fear of contagion for themselves and their family [5,6]. Secondly, in many countries, the pandemic has meant important governmental restrictions on the freedom of movement, which may have also affected mental health. A review of 24 suitably related investigations [7] revealed that the confinement situation is related to a significant increase in post-traumatic stress disorders. Finally, [8] points to an unseen media overexposure as an important variable that may explain this incremented distress. In general, the scenario is conducive to the appearance of relatively serious mental health problems, the scope of which has not yet been clearly delimited.

An objective of this article is to study the labour market effects of the COVID-19 pandemic and as such, ample evidence exists of a relationship between work status and well-being. On the one hand, various studies confirm the detrimental effects of unemployment on mental health. Some of these consider self-reported PWB, evidencing the negative effects of unemployment [9,10]. Furthermore, some job features relate to worsening mental health, such as holding a temporary job, as compared to an open-ended one [11]. Others find harmful effects such as consumption of alcohol or other drugs attributable to work-related changes [12,13]. Various meta-analyses conclude that joblessness causes mental health problems [4]. On the contrary, being employed seems to protect an individual from the presence of anxiety and depression disorders [14,15].

Thus, to the extent that the pandemic has caused job losses, a part of the psychological well-being effects being reported may be triggered by uncertainties pertaining to the working-age population. Unexpected changes in work situations, whether permanent or temporary, as a consequence of the lockdown, provide extraordinary conditions for socioeconomic analyses. The fact that in Spain numerous public and private entities sent their workers home almost overnight [16] prompted the present research on the PWB of people who were able to keep their jobs, of those who became unemployed, and of other groups who experienced new situations such as teleworking or furloughs. In this context, recent research [17] related to the pandemic shows that low-income workers, self-employed and women have decreased their teleworking productivity, with the authors proposing an additional causal route between the decrease in productivity and worse psychological well-being.

In Spain, previous studies confirm the existence of a direct relation between joblessness and worse mental health [18,19]. Additionally, a survey-based analysis carried out during the initial stages of the pandemic revealed that 18.7% and 22% of the sample had symptoms compatible with depression and anxiety, respectively [20]. However, to the best of our knowledge, no analysis exists to date that relates mental well-being in Spain with the impact of the pandemic on labour market outcomes.

The work-related changes on PWB are mediated by public policies regarding employment protection and unemployment benefits [21,22]. Among these policies, furlough schemes deserve special attention, since on many occasions workers receive pay under these schemes. Said transfers could influence the well-being of those affected. Such was the case during the COVID-19 crisis in Spain, where the so-called temporary employment regulation plans (ERTE by the Spanish acronym) became widespread. Initiating an ERTE requires a decision by the firm, and subsequent government approval. In May 2020, the force majeure option of this scheme covered up to 3.7 million workers, with its maximum duration extended from the initial date of 30 June to the end of May 2021.

Scarce literature exists as to the influence of furloughs on psychological well-being. Ref. [23] analyse the consequences of USA federal government shutdowns on furloughed employees, concluding that negative effects on well-being were associated with perceived personal resource loss. The loss, in turn, was related to decreased life satisfaction and increased work–family conflict and physical, cognitive, and emotional burnout. This sense of resource loss seems to be especially distressing for higher-level occupational groups, who perceive joblessness as a trauma to a far greater extent than other employees [23,24].

With regard to the mental health effects of telework caused by the lockdown, the available research shows conflicting results. Some analysts point out negative impacts on well-being and stress from working at home, as compared to working in the workplace [25,26]. Teleworking produces emotional effects such as isolation, irritability and worries, which increase the symptoms associated with stress, anxiety and depression. Other authors have found that teleworking may improve well-being, as it increases the ability to reconcile family life [27]. A decrease in the work-family conflict seems to depend on employees perceiving that they have some control or choice over the work location, timing and the work process itself [28]. Teleworking dictated by organisational interests, such as fixed-term contracts and involuntary part-time employment, has equivocal or negative health effects [29]. Contradictory evidence on teleworking was found more recently by [30]. They surveyed a group of Dutch workers, finding positive attitudes associated with greater efficiency or less exhaustion, and negative views linked to a sense of weakening peer ties and decreasing chances of promotion. Similar contradictory effects of teleworking were found among married women with school-aged children, who value positively their new work-life balance, while maintaining their perceived responsibility for care giving in the family [31].

Despite the ample evidence on the health effects of flexible working conditions, ref. [29] concluded a systematic review by pointing out the need for additional intervention studies, given the limitations of existing research. The COVID-19 lockdown may facilitate such an analysis, since confinement prompted a sudden growth of teleworking, which had previously been restricted to a minority portion of the working population in Spain.

Another area of research relating work situations with mental health contemplates the possibility that genders were affected differently during the COVID-19 lockdown. This was the case in the UK, where women, as well as younger people, suffered more severe consequences from the lockdown. In the case of women, that worsening mental health was related to an increase in care work, as a consequence of school closures [17]. Not surprisingly, numerous studies for United Kingdom [32] and Spain [33] demonstrate that the increase in care needs has been unequally distributed between men and women.

These findings are consistent with the mental health gender gap evidenced during the COVID-19 pandemic. Although research on psychological well-being during the pandemic reveals a general deterioration in all layers of the population [34], this deterioration is not distributed homogeneously throughout society. In Austria, ref. [35] reveal an erosion of mental health that is especially pronounced among young people, women, people without work, and with low income. Based on data obtained for the Italian population, ref. [36] conclude that young people and women will be the most affected by post-traumatic stress disorder, depression, anxiety and insomnia.

Finally, the way in which the pandemic affects men and women will also differ in relation to the labour market [37]. Using the data from several countries such as China, Italy, or the United Kingdom, ref. [38] conclude that the probability of unemployment as a consequence of the pandemic is 24% higher in women than in men. In addition, ref. [39] affirm that women will be more affected by job changes with a greater probability of moving towards either teleworking, a reduction in working hours or ultimately unemployment. These results suggest that the economic crisis prompted by the pandemic has proved especially detrimental to women, as opposed to the Great Recession, which hit men to a greater extent [40].

## 3. Hypotheses

In this article, changes in the PWB of people immediately after their labour market status was altered due to the pandemic are studied using the methodology of an ordered probit model. Moreover, given that the pandemic can be considered an external shock, any change in a working situation can be considered an exogenous variable, thus avoiding the bidirectionality issues common to this type of study. In sum, in accordance with the previous literature, the following three hypotheses are considered:

**Hypotheses** **1** **(H1)** **.** *Unemployed and furloughed people will experience a greater negative effect on their self-perceived well-being than those who are working.*

**Hypotheses** **2** **(H2)** **.** *Those who are teleworking will have a different self-perceived well-being than before the COVID-19 crisis as compared to people who continue with usual presence-based jobs in the workplace.*

**Hypotheses** **3** **(H3)** **.** *Women will experience a worse PSW as a result of the lockdown compared to men.*

## 4. Materials and Methods

Given the restrictions on mobility imposed by the Spanish government, the data was obtained through an online questionnaire accessible from 11 April to 7 May. The questionnaire was addressed to people who fulfilled three conditions: being confined in Spain, being in the labour market, and having had some paid work experience during the last 12 months. For the selection of survey participants, quota sampling was used, with gender, age and study levels as criteria variables. The combination of these three variables resulted in 18 subsamples or quotas incorporating all the different categories: three categories for education level, three categories for age and two for sex.

Online information collection methods allow higher response rates than traditional methods [41]. However, they also suffer from certain problems that should be reviewed. First, the lack of representativeness typical of all non-random sampling models. The second problem faced was the possibility of self-selection bias. In this sense, people choose whether they want to respond or not, and response rates may differ between different groups. To tackle these issues, elevation coefficients have been applied in order to return the representativeness to the specific subsamples constructed from the quotas [42]. The weighting coefficients were obtained from a sample of the last Spanish National Health Survey set in 2017. Finally, a sample of 1050 people was obtained.

The variables included in this analysis are reported in Table 1. In this model, PWB is used as the dependent variable. For measuring PWB, the General Health Questionnaire (12-item version) was included in the online survey. Originally, the answers given in the questionnaire were scored from 0 to 3. The higher the score reached in the GHQ-12, the greater the risk of suffering a mental health problem. To obtain different degrees of PWB, the mean of the responses was calculated for each person using the original GHQ-12 variable. This mean was recalculated in an ordinal variable from 1 to 3, in which the lowest category (1) included the low-risk mean, the medium category (2) included the medium-risk mean and the highest category (3) included the high-risk mean.

Regarding explanatory variables, analysing the work-related situation is of utmost importance for our study. The following situations are considered: unemployed; on furlough due to the pandemic; on leave due to other reasons; teleworking; and still working in the usual pre-pandemic workplace. Additionally, other labour-market-related variables are included (years of work experience; occupational group; economic sector of the current job or the last job for those unemployed; and income level), as well as other variables related to confinement conditions that may prove important for analysing the effects of the lockdown (such as square metres per capita in the dwelling, people at risk of COVID-19 in the household, the total number of confined people in the household, minors in the household during confinement and dwellings with patio). Finally, we included individual variables (age, sex, education level, disability, marital status and being born in Spain).

Regarding methods, an ordered probit model was used to estimate the relationship between mental health (our ordinal dependent variable) and a set of socioeconomic characteristics for individuals that could affect their mental health, paying special attention to the impact of their labour market status. Basically, an ordered probit model is a generalisation of a probit in which there are more than two possible outcomes for an ordinal dependent variable (see, for example [43]).

However, one of the risks of this type of study is the assumption that the relation between the labour market status and PWB is unidirectional, when in fact, it could be bidirectional. Hence, unemployed people could be in a worse mental health situation, but people with psychological distress may face a higher probability of being unemployed than those people with better mental health [44].

The authors followed several strategies to cope with the foregoing problem. To control this individual heterogeneity, some authors have proposed fixed effect models via the use of longitudinal data [9] or controlling the health selection effect via the use of Instrumental Variables for unemployment [45,46]. Another strategy is to analyse the health of people immediately after losing their jobs due to firm closures [47,48]. The objective of this latter strategy is to introduce an exogenous cause of unemployment (e.g., the closure of a factory), with the aim of preventing the cause of unemployment from directly affecting the health of the person. In this regard, ref. [49] adopted a strategy for overcoming the simultaneity problem in the Spanish case. These authors restricted the analysis to the construction sector in Spain, which, due to the great recession that began in 2008, lost up to 60% of its previous employment level.

In line with these strategies, this study analysed the relationship between the PWB and labour market status immediately after the special circumstances created by the COVID-19 pandemic. As already mentioned, the widespread employment-situation changes involved either a move from employment to unemployment or furloughs or a shift from ordinary employment to telework. This situation is external to the person, so the objective is to leverage this situation in order to reduce the bidirectional problems inherent in the analysis.

## 5. Results

Table 2 includes the distribution of all variables in the analysis as per the different categories of the work-related situation. It is noteworthy that, within the descriptive results, the percentage of women is higher than men for unemployed, furloughed, on leave (other reasons), or teleworking individuals. These data are consistent with the literature that has studied the pandemic’s impact on the employment situation of men and women [39]. Also worth mentioning is the large percentage of people with a high level of education among those who are teleworking since the outbreak of the pandemic, this also being corroborated by the literature on the subject [33].

The results of the estimations are shown in Table 3. The second column presents the results of a general model, while separate results for men and women are shown in the third and fourth columns. Regarding the general results, the work-related situation variable offers results significantly different from zero. Thus, people without jobs, those on furlough, and those who are teleworking present a greater risk of PWB than people who continue working in their usual pre-pandemic workplace. This suggests that the risks of getting COVID, which could affect people who continue in the usual workplace, is less significant than job-related risks.

Moreover, given that the main objective of this study is to analyse how changes in labour market status due to the pandemic have affected PWB, the marginal effects for the work-related situations have also been calculated. These effects (Table 4) show that, although the detrimental impact of teleworking is lower than that of being unemployed or furloughed due to COVID-19, working in the same place as prior to the lockdown is the most beneficial option for PWB. For the complete sample, being unemployed increases the probability of being in a high-risk situation by 12.64%. Having been out of work due to the pandemic increases the probability of being at high PWB risk by 15.19%.

## 6. Discussion

According to the theory of latent employment functions [50], the consequences of unemployment can be interpreted in contrast to the functions of having a job: income, the imposition of a daily routine, establishment of personal ties and shared experiences, the possession of objectives and purposes that transcend the individual, ascription to status and the obligation to maintain a certain level of activity. It is the absence of these work functions, one of the most evident effects of the COVID-19 pandemic, that could be contributing towards mental health problems. The interruption of employment that took place for some people after the lockdown in March 2020 is likely to be the factor explaining the increased risk in psychological well-being. Conversely, continuing work at the same workplace (and to a lesser extent, teleworking), appears to be protecting people’s PWB. Finally, as already mentioned, teleworking increases the risk by 8.36% with respect to people who continue to work at their usual workplace instead of at home.

Thus, Hypothesis 1 and Hypothesis 2 of this article are confirmed by the results: first, unemployed and furloughed people have experienced a greater negative effect of the lockdown on their self-perceived well-being than those who are employed; second, among those people who have continued to work, teleworkers have experienced less self-perceived well-being than people who continue working at the same place as before the COVID-19 crisis.

Regarding other work-related variables, occupation, experience and economic sector, significant coefficients were revealed for some of the categories. Using groups from the National Classification of Occupations, all three models show that managers, directors and senior officials (reference group) seem to have a relatively high risk of worsening self-perceived well-being. As compared to this group, people whose current or last occupation was a professional one or who were in administrative and secretarial occupations present less risk. Coefficients pertaining to the other two socioeconomic variables indicate, first, that people with more years of work experience are better off. With regard to work experience, it could be a variable related to job stability and with a lower probability of being unemployed. People with more experience may be those who have continued their work after the pandemic started. Second, those whose current or last job was in the primary sector present a lower risk than those in the secondary sector. As regards the economic sector, people in the primary sector may be less likely to be among those with a higher risk of mental health given that they have a greater probability of maintaining their work habits. This is because the activities of the primary sector were part of the essential activities that remained active during the lockdown.

Given the situation of confinement imposed by the authorities, the variables related to the dwelling where persons were confined are particularly relevant. In this sense, living alone during confinement is detrimental to PWB. The risk decreases as the number of people with which the confinement was passed increases. Moreover, being confined in a dwelling with a recreational space such as a patio decreases the probability of belonging to the most severely impacted group. Furthermore, the space available per person appears to be an important variable: more square metres per capita seem to diminish the risk of worsening well-being.

Regarding the ordered probit coefficients corresponding to individual features, results suggest that the younger a person is, the higher the probability of obtaining a high-risk score. Marital status also influences psychological well-being: singles have had greater PWB during the pandemic and confinement than married people. Finally, being a woman increases the probability of suffering deterioration in well-being. This result is in line with the previous literature regarding the consequences of the pandemic and proves the third hypothesis of this study.

Due to the latter hypothesis, the results of the model are presented as differentiated by gender. As already mentioned, the third and fourth columns in Table 4 are dedicated to the results of the ordered probit model for men and women, respectively. Regarding the work-related situation, results do not differ from those obtained with the complete sample. Both unemployed men and women and those on furlough due to the pandemic are at higher risk than those who continue working at the usual workplace, thereby confirming the robustness of the previous results for both sexes. Interestingly, regarding people who telework, the category is only significant (and, again, with a positive sign) for women. Neither do the results found in men nor in women vary with those obtained in the general sample for age and marital status. However, when analysing men and women separately, the variable of income becomes significant. Among men, the higher the level of income, the lower the risk of harm to PWB and, conversely, among women, higher levels of income correspond to higher levels of risk.

It is worth mentioning that those variables related to household production and historically associated with women present significant coefficients. Living with someone with a risk of being infected by COVID-19 negatively affects mental health, but only for women. Living with minors negatively affects women also and, rather surprisingly, positively affects men. In other words, women who are confined with minors have a greater risk of being in the highest risk group of people, while the same situation for men increases their probability of belonging to the lowest risk group.

In general, living alone affects women more than men. The analysis by occupational group shows that women who are professionals and those in the administrative and secretarial occupations group present less risk. Likewise, in the case of men in occupations such as catering, protection and sales, as well as other low-skilled workers, there appears to be a lower risk of worsening their self-perceived well-being. The square metres per capita of the dwelling and the years of work experience are also significant for women and the coefficients present, as in the case of the complete sample, negative signs, that is, the explanation would be analogous to the one described previously: more space and work experience seem to lower the risk. Finally, the activity sectors are only significant for men and both those in the primary and tertiary sectors have a lower risk than men in the secondary sector.

Regarding the work situation, marginal effects separated by men and women are shown in Table 4. This disaggregation of the sample by gender makes it possible to analyse, first of all, how the effect of the work situation on mental health differs for men and women. Unemployment affects men more than women. Hence, the probability that an unemployed person is in the highest risk range increases by 13.44% for a man. In the case of an unemployed woman, the risk is also higher compared to people who continue to work, but its effect decreases to 8%. Similarly, the probability that an unemployed person is in the lowest risk range is lower for men. In summary, in the complete sample those work situations that negatively affect mental health impact men more than women.

This effect of the unequal impact of working conditions among men and women has already been described in the literature. The explanation could lie in the degree of connection maintained by a person with the labour market. Ref. [51] conclude, based on data with the Spanish population, that the Great Recession of 2008 influenced men and women differently. Thus, the increase in psychological disorders during the crisis years was greater in men than in women. According to these authors, this effect could depend on the different “gender regimes”. While in Southern Europe male identity appears to be linked to employment, women still seem to fulfil a role more associated with care work. On the contrary, in societies where the relationship between men and women with respect to the labour market is similar, there are no differences in the way in which labour variables affect both sexes [52]. In summary, the greater male connection with the labour market (in terms of a higher rate of full-time employment or permanent contracts) makes them more vulnerable to job loss. Thus, the greater stability of men in the Spanish labour market may explain this different effect of the labour situation between the sexes.

The lesser connection with the labour market for women and the higher rates of temporary employment for women could also be associated with the care work carried out by men and women. In Spain, the distribution of tasks is very different depending on gender (in 2016, the Spanish Statistical Office reported that weekly hours spent for childcare and education were 23 for men and 38 for women, and the hours dedicated to domestic work were 11 for men and 20 for women). This could explain the result obtained in Table 4 that indicates that having been confined with minors and with a population at risk of suffering COVID-19 also negatively affects PWB, although in these last two situations, only in the case of women.

The role that society gives to women in Spain, although it has evolved, continues to be related to reproduction and care work. This could be related to the negative impact that the latter have on women, as revealed from the analyses carried out in this research. This greater care workload could explain, in the first place, the lower percentage of permanent and full-time contracts for women and, consequently, a partial or complete separation from the labour market, which ultimately explains the lower impact that the lockdown had on women in Spain.

Additionally, this lesser connection with the labour market could also explain the different results regarding income levels by sex found in the ordered probit model. Among men, those with middle and high incomes are less likely to suffer a mental health problem than those with low incomes, while among women the opposite happens, with those with high incomes presenting a greater risk. Perhaps the lower linkage of women with the labour market intensifies among low-income women. This reduced connection could be causing high-income women, who hold more stable jobs and greater social recognition, to see greater threats in the situation caused by COVID-19 than those women with lower incomes, who hold more precarious and unstable jobs and whose contractual relationship will most likely be a temporary one.

## 7. Conclusions

While the physical effects of COVID-19 are widely recognized, the relationship between employment status and mental health during confinement has been studied less. Here, the results confirm that changes in employment status due to COVID-19 have had a significant impact on PWB. Unemployed people and those who have been unable to work due to the lockdown have a greater probability of reduced self-perceived well-being. This conclusion is consistent with other evidence emerging in the literature. Furthermore, teleworking also has an adverse impact on self-perceived well-being, but to a lesser extent than being furloughed or unemployed. In sum, the data have confirmed the three hypotheses proposed in the article.

The results obtained in this research point to the importance of including the labour market as a priority area for recovery once the most acute phase of the pandemic has been overcome. Recovering the jobs lost during the pandemic should be on the agenda of all governments. The labour market is presented as one of the main providers of well-being, and its implications could go as far as emotional well-being. In this context, the knowledge accumulated over the years should result in measures to incorporate health assistance as an essential part of the needs of unemployed people. Furthermore, these needs should be contemplated not only from an employment policy perspective, but also as part of a broader system that incorporates the mental health care of the unemployed as part of more general public health policies.

This research is not without limitations. The survey conducted is a single cross-section one with limited sample size. The conclusions would possibly be more robust with a longitudinal database. However, as mentioned earlier, the external shock as a result of the pandemic served as an opportunity to limit the possible bias of this type of data collection. Furthermore, the use of weights from the Spanish National Health Survey confers representativeness to the sample. It is expected that, in the future, these analyses can be completed and complemented with a longitudinal analysis that will give more robustness to the conclusions achieved by this research.

## Figures and Tables

**Table 1:** Variables included in the analysis.

Variable	Rank
Mental well-being (dependant variable)	1 low level of risk; 2 medium level of risk; 3 high level or risk
Labour situation	1 if the person is unemployed; 2 if the person is on furlough due to the pandemic; 3 if the person is on leave due to other reasons; 4 if the person is teleworking; 5 if the person still works at the usual pre-pandemic workplace
Sex	1 man; 2 woman
Age	Continuous variable
Education level *	1 low level; 2 medium level; 3 university level
Income	1 if the person has a net monthly income below 1200 €; 2 if the person has an income of 1200 to 2000 €; 3 if the person has an income of 2000 € upwards
Disability	1 if the person is not disabled; 2 otherwise
Marital status	if the person is married; 2 if the person is separated or divorced; 3 if the person is single; 4 if the person is widowed
People at risk of COVID-19 in the household	1 if the person lives with someone at risk of infection by COVID-19; 0 otherwise
M^2^ per capita in the dwelling	Continuous variable
Spain as a country of birth	1 if the person was born in Spain; 0 otherwise
Number of confined people in the household	1 if the person lives alone during confinement; 2 if two people live together; 3 if three people live; 4 if four people live; 5 if five people or more live together during confinement
Minors in the household	1 if the person lives with a minor during confinement: 0 otherwise
Dwelling with a patio	1 if people do not have a patio in their dwelling; 2 if the person has a patio
Occupational group	1 directors and managers; 2 scientific and intellectual professionals; 3 support professionals; 4 accounting and administrative employees; 5 catering, protection and sellers; 6 other lower-skilled workers
M^2^ per capita in the dwelling	Continuous variable
Years of work experience	Continuous variable
Economic sector	1 primary sector; 2 secondary sector; 3 tertiary sector

* Low-level education includes people with primary education or less. The middle level includes people who completed high school or vocational education and training. The third level includes people with a university degree.

**Table 2:** Distribution of variables depending on work-related situations.

	Unemployed	Furloughed Due to COVID-19 Lockdown	On Leave for Other Reason	Teleworking	Working at the Usual Pre-COVID-19 Workplace	Total
**Sample size**	116	168	42	462	262	1 050
**Sex** (%)						
Men	41.38	37.50	28.57	39.39	45.80	40.48
Women	58.62	62.50	71.43	60.61	54.20	59.52
**Age** (mean)	41.46	44.02	48.57	44.92	45.34	44.65
**Educational level** (%)						
Low Level	12.93	16.07	7.14	1.30	11.07	7.62
Medium Level	33.62	38.69	42.86	11.90	36.64	26.00
High Level	53.45	45.24	50.00	86.80	52.29	66.38
**Income** (%)						
Low Level	68.10	47.31	28.57	12.64	28.08	28.83
Medium Level	23.28	41.32	45.24	44.01	43.46	41.19
High Level	8.62	11.38	26.19	43.36	28.46	29.98
**Marital status** (%)						
Married	30.43	45.51	57.14	53.36	58.78	51.10
Separated	15.65	10.78	14.29	9.33	8.78	10.32
Single	53.04	41.92	23.81	37.09	32.06	37.82
Widow/er	0.87	1.80	4.76	0.22	0.38	0.76
**Household members at risk of COVID** (%)	47.41	40.48	61.90	30.30	30.15	35.05
**Spain as country of birth** (%)	93.97	97.02	100.00	94.81	96.18	95.62
**Confined people in dwelling** (mean)	2.54	2.73	2.88	2.70	2.73	2.70
**Minors** (%)	33.62	37.50	45.24	38.96	40.84	38.86
**Dwelling with patio** (%)	23.28	17.96	21.43	15.03	21.37	18.26
Occupational group * (%)						
1	3.51	4.17	2.56	3.03	3.05	3.25
2	28.95	22.62	33.33	70.13	33.97	47.56
3	11.40	19.64	12.82	14.07	13.36	14.45
4	15.79	8.33	10.26	11.26	9.16	10.72
5	20.18	29.76	33.33	1.30	22.52	14.45
6	20.18	15.48	7.69	0.22	17.94	9.57
**M^2^ per capita** (mean)	38.68	34.44	34.65	38.23	37.47	37.34
**Work experience** (mean. years)	14.76	19.04	22.52	19.93	21.70	19.76
**Economic sector** (mean)						
Primary	3.45	2.98	0.00	1.95	3.82	2.67
Secondary	12.93	14.29	19.05	11.26	20.99	14.67
Tertiary	83.62	82.74	80.95	86.80	75.19	82.67

* The different categories of the occupational groups correspond to the following: 1 Directors and managers; 2 Scientific and intellectual professionals; 3 Support professionals; 4 Accounting and administrative employees; 5 Catering, protection and sellers; 6 Other lower-skilled workers.

**Table 3:** Ordered probit results.

		General Estimation				Estimation for Men				Estimation for Women		
*n*		1050				425				625		
**Working situation** (ref: working at the usual workplace)												
Unemployed		0.84437 (0.21954)		***		1.07112 (0.31854)		***		0.51415 (0.27345)		*
Furloughed due to the COVID-19 lockdown		0.96063 (0.22277)		***		1.11323 (0.30056)		***		0.63360 (0.24929)		**
On leave for other reasons		0.17952 (0.40012)				0.75994 (0.56575)				−0.49211 (0.52670)		
Teleworking		0.62452 (0.22318)		***		0.40694 (0.25144)				0.53651 (0.26023)		*
**Gender** (ref: men)												
Women		0.35135 (0.16010)		**								
**Age**		−0.02621 (0.01169)		**		−0.04544 (0.01783)		**		−0.03127 (0.01572)		*
**Education level** (ref: low level)												
Medium level		−0.16840 (0.19569)				−0.33784 (0.22722)				0.04477 (0.27559)		
High level		−0.19317 (0.22081)				−0.25101 (0.28731)				−0.33114 (0.29835)		
**Income** (ref: low level)												
Medium level		−0.12593 (0.20815)				−0.43807 (0.26095)		*		0.35100 (0.23761)		
High level		−0.08224 (0.26682)				−0.56825 (0.32882)		*		0.53434 (0.28456)		*
**Disability** (ref: no)		0.48560 (0.43536)				0.65574 (0.72492)				0.36230 (0.36057)		
**Marital status** (ref: married)												
Separated or divorced		−0.07292 (0.30288)				0.33120 (0.33514)				−0.20964 (0.32113)		
Single		−1.2446 (0.23572)		***		−0.96734 (0.26939)		***		−1.12968 (0.26501)		**
Widow/widower		−0.57264 (0.94144)				−1.14898 (1.35795)				0.72199 (0.47620)		
**Household members at risk of COVID-19** (ref: no)		0.24100 (0.16540)				−0.12584 (0.20327)				0.59719 (0.21057)		***
**Spain as a country of birth** (ref: no)		0.25263 (0.37677)				−0.22694 (0.46492)				0.83380 (0.46935)		*
**Number of people confined in the household** (ref: person living alone)												
Two people		−1.5367 (0.37031)		***		−0.75429 (0.41316)		*		−2.08436 (0.50845)		***
Three people		−1.75079 (.51680)		***		0.09374 (0.51602)				−2.87277 (0.65740)		***
Four people		−2.00972 (0.53839)		***		−0.22890 (0.56108)				−2.97841 (0.68399)		***
Five or more		−1.97991 (0.65115)		***		0.04788 (0.68921)				−3.58189 (0.78616)		***
**Minors in the household** (ref: no)		−0.00836 (0.25626)				−0.74595 (0.30690)		**		0.69000 (0.24046)		***
**Dwelling with a patio** (ref: no)		−0.36685 (0.18331)		**		−0.35111 (0.24115)				−0.49659 (0.24993)		**
**Occupational group** (ref: directors and managers)												
Scientific and intellectual professionals		−0.61117 (0.29608)		**		−0.38082 (0.38284)				−0.89385 (0.45075)		**
Support professionals		−0.66527 (0.32156)		**		−0.33565 (0.40105)				−1.25528 (0.51564)		**
Accounting and administrative employees		−0.91837 (0.36787)		**		−0.32109 (0.38822)				−1.0982 (0.54458)		**
Catering, protection and sellers		−0.49537 (0.33185)				−0.90008 (0.44374)		**		−0.26511 (0.47890)		
Other lower-skilled workers		−0.37783 (0.34736)				−0.74540 (0.41828)		*		−0.36606 (0.60137)		
**M^2^ per capita in dwelling** (continuous variable)		−0.02103 (0.00805)		**		−0.00139 (0.00800)				−0.02655 (0.01066)		**
**Years of work experience** (log continuous variable)		−0.40894 (0.17989)		**		0.19020 (0.24229)				−0.57945 (0.19978)		***
**Economic sector** (ref: secondary)												
Primary	−1.02120 (0.48153)		**		−1.38499 (0.52273)		**		−0.25981 (0.72239)			
Tertiary	−0.34072 (0.22626)				−0.62145 (0.22238)		**		0.12946 (0.37795)			
**Cut1**	−5.67907 (1.06473)		***		−4.14714 (1.10960)		***		−6.43643 (1.46119)		***	
**Cut2**	−3.70860 (1.05566)		***		−2.03827 (1.10986)		*		−4.23000 (1.39009)		***	

Levels of significance: * *p* < 0.05; ** *p* < 0. 01; *** *p* < 0.001.

**Table 4:** Marginal effects for work-related situations (ref: working at the usual workplace).

	General Estimation		Estimation for Men		Estimation for Women	
**Unemployed**						
Low Risk	−0.2445	***	−0.3095	***	−0.1207	*
Medium Risk	0.1180	***	0.1751	***	0.0398	
High Risk	0.1264	***	0.1344	**	0.0809	*
**Furloughed due to COVID-19 lockdown**						
Low Risk	−0.2726	***	−0.3195	***	−0.1462	**
Medium Risk	0.1207	***	0.1764	***	0.0426	
High Risk	0.1519	***	0.1430	***	0.1036	**
**On leave for other reasons**						
Low Risk	−0.0557		−0.2291		0.1260	
Medium Risk	0.0369		0.1500	*	−0.0733	
High Risk	0.0187		0.0791		−0.0526	
**Teleworking**						
Low Risk	−0.1867	***	−0.1260		−0.1256	**
Medium Risk	0.1030	***	0.0925		0.0405	
High Risk	0.0836	**	0.0335		0.0850	**

Levels of significance: * *p* < 0.05; ** *p* < 0.01; *** *p* < 0.001.

## Data Availability

The data presented in this study is available on request from the corresponding author. The data are not publicly available due to is personal information.
